# Expression of Retinoid Acid Receptor-Responsive Genes in Rodent Models of Placental Pathology

**DOI:** 10.3390/ijms21010242

**Published:** 2019-12-29

**Authors:** Alexander Mocker, Marius Schmidt, Hanna Huebner, Rainer Wachtveitl, Nada Cordasic, Carlos Menendez-Castro, Andrea Hartner, Fabian B. Fahlbusch

**Affiliations:** 1 Department of Pediatrics and Adolescent Medicine, Friedrich-Alexander University Erlangen-Nuremberg, 91054 Erlangen, Germany; alexmocker@t-online.de (A.M.); mschmidtdav@gmail.com (M.S.); Carlos.Menendez-Castro@uk-erlangen.de (C.M.-C.); andrea.hartner@uk-erlangen.de (A.H.); 2 Department of Gynaecology and Obstetrics/Comprehensive Cancer Center Erlangen-EMN, Friedrich-Alexander University Erlangen-Nuremberg, 91054 Erlangen, Germany; hanna.huebner@uk-erlangen.de; 3 Department of Nephrology and Hypertension, Friedrich-Alexander University Erlangen-Nuremberg, 91054 Erlangen, Germany; Rainer.Wachtveitl@uk-erlangen.de (R.W.); Nada.Cordasic@uk-erlangen.de (N.C.)

**Keywords:** RARRES, chemerin, placenta, IUGR, PE, eNOS-knockout, CmklR1, IL-11, low protein diet, pregnancy

## Abstract

In humans, retinoic acid receptor responders (RARRES) have been shown to be altered in third trimester placentas complicated by the pathologies preeclampsia (PE) and PE with intrauterine growth restriction (IUGR). Currently, little is known about the role of placental Rarres in rodents. Therefore, we examined the localization and expression of Rarres1 and 2 in placentas obtained from a Wistar rat model of isocaloric maternal protein restriction (E18.5, IUGR-like features) and from an eNOS-knockout mouse model (E15 and E18.5, PE-like features). In both rodent models, Rarres1 and 2 were mainly localized in the placental spongiotrophoblast and giant cells. Their placental expression, as well as the expression of the Rarres2 receptor chemokine-like receptor 1 (*CmklR1*), was largely unaltered at the examined gestational ages in both animal models. Our results have shown that RARRES1 and 2 may have different expression and roles in human and rodent placentas, thereby underlining immanent limitations of comparative interspecies placentology. Further functional studies are required to elucidate the potential involvement of these proteins in early placentogenesis.

## 1. Introduction

Our previous human studies indicated a dysregulation of the tumor suppressor genes retinoic acid receptor responsive proteins (retinoic acid receptor responders, RARRES) 1 and 2 in the third trimester placentas complicated by preeclampsia (PE) and PE conjoined with intrauterine growth restriction (IUGR) [1,2]. We observed an induction of RARRES1 expression in primary villous cytotrophoblasts isolated from PE and PE/IUGR placentas with a concomitant increase in RARRES1 syncytial staining. RARRES2 mRNA expression, on the contrary, seemed reduced, yet unaltered at the protein level in third trimester villous placental samples [1]. These results are controversial, as others have found increased RARRES2 protein expression in samples from total placentas in pregnancies complicated by PE [3]. Furthermore, we had previously determined that RARRES1 and 2 were located in distinct functional placental compartments [1]. RARRES1 (also known as Tazarotene-induced gene 1 (TIG1), Latexin-like (LXNL), or Phorbol Ester-induced gene 1 (PERG-1) [4]) was located in human villous and extravillous trophoblast cells (EVT) [1], while RARRES2 (also known as chemerin, HP10433, and TIG2 [5]) was specifically expressed in human placental EVTs [1]. In contrast, Garces et al. described an additional placental RARRES2 expression in cytotrophoblasts and Hofbauer cells [6].

RARRES1 stimulates the expression of antioxidant enzymes, inhibits angiogenesis, and stimulates autophagy via mTOR [7]. In line with its proposed tumor suppressor function [8,9,10], RARRES1, along with RARRES2, was reduced in choriocarcinoma [2] and its expression was also significantly reduced in certain choriocarcinoma cell lines (i.e., Jeg-3 and BeWo) [1].

While RARRES1 is located intracellularly [10,11], RARRES2 is a secreted adipocytokine that requires activation of its pro-form by proteolytic cleavage to exert its functions via chemokine-like receptor 1 (CMKLR1, ChemR23) [12,13]. Wang et al. [3] were able to show that RARRES2 exerts anti-inflammatory functions by inducing endothelial nitric oxide synthase (eNOS) expression in human umbilical vein endothelial cells (HUVECs) and by significantly decreasing TNF-α-induced nuclear factor (NF)-kappa B, and vascular cell adhesion molecule (VCAM)-1 production [3]. RARRES2 further modulates chemotaxis and activation of dendritic cells and macrophages via CMKLR1 [14,15], which is expressed in various leukocyte populations [16].

Pregnancy represents a state of constant metabolic adaptation and increased inflammation. In this respect, IUGR and PE represent two extreme gestational disturbances [17,18,19]. In PE the production of placental inflammatory cytokines [18,19] is increased. It is known that adipocytokine and interleukin signaling interact [20,21,22]. Recently IL-11, a member of the IL-6 family also known as adipogenesis inhibitory factor (AGIF) [23], has been found by others to be upregulated in PE and leads to inflammation and preeclampsia-like features in mice [24]. Treatment of mice with IL-11 negatively affects placentation, including trophoblast invasion and spiral artery remodeling, a key process in the pathogenesis of human PE [24,25]. IL-11 further increases systolic maternal blood pressure and leads to PE-like proteinuria in dams [24,25]. Mice with an eNOS-deficiency (eNOS^−/−^ [26,27,28,29]) display PE-like features (e.g., vascular placental impairment [19,27,30] and an increased inflammatory state [31,32,33,34]).

To confirm this, we tested placental IL-11 expression in these mice. Moreover, we analyzed Rarres expression in second and third trimester placenta of eNOS^−/−^ mice, because Garces et al. detected a maximum of placental Rarres2 expression at this gestational age in rodents [6].

To expand our findings from third trimester human placenta [1], we investigated Rarres1, Rarres2, and Cmklr1 expression in third trimester rodent placenta. Moreover, we analyzed the influence of maternal protein restriction in rats (IUGR-like features [35,36]) on placental Rarres1 and 2 expression. We additionally compared placentas in the context of fetal sex, given the differences in Rarres2 expression that were already described by Watts et al. [37] for male and female fetuses.

## 2. Results

### 2.1. Auxology

Animal data are displayed in Table 1. Maternal protein restriction led to a significant decrease in fetal weight (*p* = 0.03) and a significant increase in placental/fetal ratio (*p* = 0.03) at E18.5 in rats. This had no significant influence on placental weight (*p* = 0.11). In our eNOS^−/−^ mice, fetal and placental weights were examined at E15 and E18.5. The animals showed a significant decrease of fetal weight at both time points compared to wildtype controls (*p* < 0.001). Mouse placental weights were significantly decreased at E18.5 (*p* = 0.006), with a similar trend at E15 (*p* = 0.08). The placental-to-fetal weight ratio was unaffected by eNOS deficiency (Table 1).

### 2.2. Localization of Rarres1 and 2

**Figure 1:**
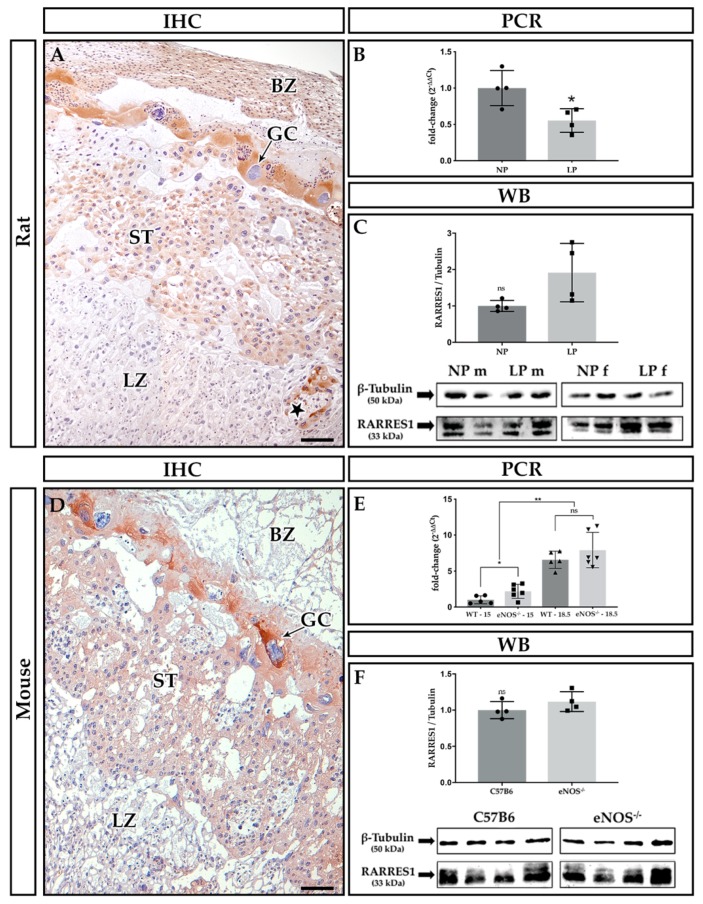
Rarres1 expression in rat and mouse placenta. (**A**–**C**) Rat placenta, (**D**–**F**) mouse placenta. (**A**,**D**) Immunohistochemical (IHC) stains of methyl Carnoy-fixed placental paraffin sections. Abbreviations: GC = giant cell, BZ = basal zone, ST = spongiotrophoblast, LZ = labyrinth zone, star = glycogen cells. The bar equals 100 µm. (**B**) Maternal protein restriction rat model: placental *Rarres1* mRNA expression on E18.5 (*p = 0.03, Mann-Whitney *U*-Test, *n* = 4 NP/LP dams with 6 pups each). E) eNOS^−/−^ mouse model: placental *Rarres1* mRNA expression on E15 and E18.5 (* *p* = 0.03, ** *p* = 0.008 for C57BL/6 and *p* = 0.002 for eNOS^−/−^, ns: *p* = 0.66, Mann-Whitney *U*-Test, WT: *n* = 5 dams, eNOS^−/−^: *n* = 6 dams with 2 pups each). C + F) Analysis of Rarres1 protein expression versus β-Tubulin housekeeper by Western blotting (WB, Rat: ns: *p* = 0.057, Mann-Whitney *U*-Test, *n* = 4 NP/LP dams with *n* = 2 pups each, E18.5; Mouse: ns: *p* = 0.20, Mann-Whitney *U*-Test, *n* = 4 C57B6 and eNOS^−/−^ dams per group with *n* = 1 pup each, E18.5). Abbreviations: LP = low protein diet, NP = normal protein diet in the rat IUGR model with m = male fetus, f = female fetus; C57B6 = C57BL/6 wild type (WT) control strain, eNOS^−/−^ = preeclampsia (PE)/intrauterine growth restriction (IUGR) model eNOS knockout mouse, ns = not significant. RARRES = retinoic acid receptor responders.

**Figure 2:**
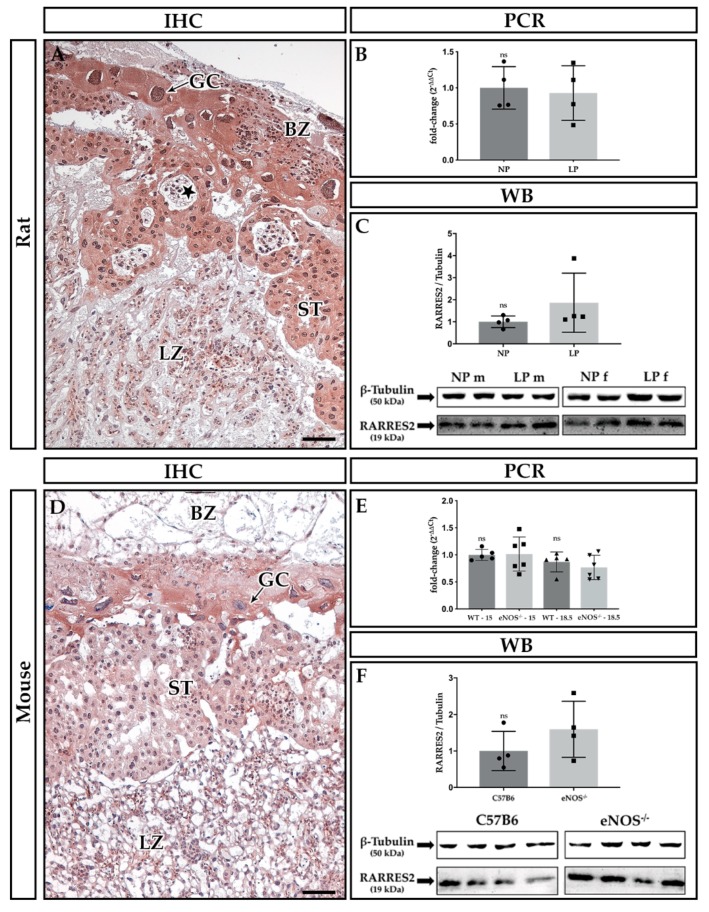
Rarres2 expression in rat and mouse placenta. (**A**–**C**) Rat placenta, (**D**–**F**) mouse placenta. (**A**,**D**) Immunohistochemical (IHC) stains of methyl Carnoy-fixed placental paraffin sections. Abbreviations: GC = giant cell, BZ = basal zone, ST = spongiotrophoblast, LZ = labyrinth zone, star = glycogen cells. The bar equals 100 µm. (**B**) Maternal protein restriction rat model: placental *Rarres2* mRNA expression on E18.5 (ns: *p* = 0.89, Mann-Whitney *U*-Test, *n* = 4 NP/LP dams with 6 pups each). E) eNOS^−/−^ mouse model: placental *Rarres2* mRNA expression on E15 and E18.5 (E15: ns: *p* = 0.31, E19: ns: *p* = 0.66, Mann-Whitney *U*-test, WT: *n* = 5 dams, eNOS^−/−^: *n* = 6 dams with 2 pups each). C + F) Analysis of Rarres1 protein expression versus β-Tubulin housekeeper by Western blotting (WB, Rat: ns: *p* = 0.10, Mann-Whitney U-Test, *n* =4 NP/LP dams with *n* = 2 pups each, E18.5; Mouse: ns: *p* = 0.49, Mann-Whitney *U*-Test, *n* = 4 C57B6 and eNOS^−/−^ dams per group with *n* = 1 pup each, E18.5). Abbreviations: LP = low protein diet, NP = normal protein diet in the rat IUGR model with m = male fetus, f = female fetus; C57B6 = C57BL/6 wild type (WT) control strain, eNOS^−/−^ = PE/IUGR model eNOS knockout mouse, ns = not significant.

Representative images of Rarres1 and 2 immunohistochemical (IHC) stains are given in Figure 1A,D and Figure 2A,D, respectively. Both proteins shared similar localization in functional placental compartments. In contrast to Rarres1 (cytoplasmic stain, Figure 1), Rarres2 (Figure 2) additionally showed nuclear staining. IHC did not reveal species differences between rat (Figure 1A and Figure 2A) and mouse (Figure 1D and Figure 2D) placentas regarding Rarres1 and 2 localization. Positive staining was mostly present in the cytoplasm of trophoblast giant cells (GC) and spongiotrophoblasts (ST) of rat (Figure 1A) and mouse (Figure 1D) placentas at E18.5. In mice, we did not note differences in Rarres1 and 2 staining in comparison to E15 (data not shown). We additionally found positive staining for both proteins in the yolk sac, decidual stroma, and the umbilical cord lining membrane (data not shown). Glycogen cells and the labyrinth zone (LZ) stained negative for Rarres1 and 2.

### 2.3. Expression Analyses of Rarres1/2, CmklR1 Receptor, and IL-11

We detected a small but significant decrease of placental *Rarres1* mRNA expression in our maternal protein restriction rat model at E18.5 (*p* = 0.03, Figure 1B). Sex did not show any significant influence on the expression of *Rarres1*, *2* and *CmklR1* mRNA expression levels (Table 2). In contrast to the rat, we could determine significant differences in *Rarres1* mRNA expression between eNOS^−/−^ mice and C57BL/6 wildtype controls at E15 (*p* = 0.03) but not on E18.5 (*p* = 0.66) (Figure 1E). However, a 3.6-fold (eNOS^−/−^) and 6.5-fold (C57BL/6) temporal increase of placental *Rarres1* mRNA expression was detected from E15 to E18.5 (*p* = 0.008 for C57BL6 mice and *p* = 0.002 for eNOS^−/−^ mice, Figure 1E). Western blot analysis did not reveal significant differences in placental Rarres1 protein expression E18.5 in both animal models (rat: *p* = 0.057, mouse: *p* = 0.20, Figure 1C,F).

Placental *Rarres2* mRNA (Figure 2B,E) and protein expression (Figure 2C,F) was neither affected by maternal protein restriction in the rat (PCR: *p* = 0.89, WB: *p* = 0.10, Figure 2B,C), nor eNOS^−/−^ in the mice (PCR: E15: *p* = 0.31, E19: *p* = 0.66, WB: *p* = 0.49, Figure 2E,F). Also, the expression of *Rarres2* remained unchanged from E15 to E18.5 in the mouse (*p* = 0.31 for C57BL6 mice and *p* = 0.13 for eNOS^−/−^ mice, Figure 2E).

**Figure 3:**
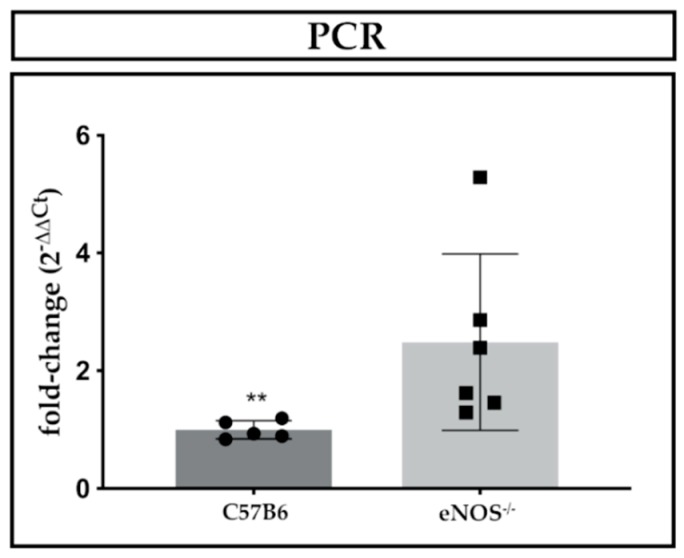
Placental *IL-11* mRNA expression in eNOS^−/−^ mice on E15. (** *p* = 0.004, Mann-Whitney *U*-test, WT: *n* = 5 dams, eNOS^−/−^: *n* = 6 dams with 2 pups each) Abbreviations: C57B6 = C57BL/6 wild type (WT) control strain, eNOS^−/−^ = PE/IUGR model knockout mouse.

Placental *CmklR1* expression was unchanged by maternal protein restriction in the rat and eNOS^−/−^ in the mouse. Gestational age seemed to have no significant influence on *CmklR1* expression in the mouse (Table 3). However, a significant increase of placental interleukin 11 (*IL-11*) mRNA expression at E15 (2.3-fold, *p* = 0.004, Figure 3) was observed in eNOS^−/−^ mice compared to controls. No such change in *IL-11* expression was noted at E18.5 (*p* = 0.99).

## 3. Discussion

Summarizing our findings, we demonstrate a sufficient induction of intrauterine growth restriction in both rodent models, as determined by fetal weight reduction, when compared to the respective controls. Rarres1 and 2 were pre-dominantly located in the spongiotrophoblast and giant cells of both rat and mouse placenta. In the rat, no consistent regulation of Rarres1/2 was detected under maternal protein restriction. Similarly, we did not find changes in *Rarres1* and *2* mRNA or protein expressions in eNOS^−/−^ mice. In the mouse, we observed a temporal increase in placental *Rarres1* mRNA expression from E15 to E18.5 independent of their genotype. Moreover, we found an IL-11 induction at E15 in the eNOS^−/−^ mice, suggestive of increased placental inflammation, a common feature of human PE [24].

### 3.1. Expression of Rarres1 in Rodent Placenta

Based on its localization in the human placenta, we have previously hypothesized that RARRES1, as a tumor-suppressor gene, might slow down invasion and migration of EVTs in terminal placentas and that it might regulate proliferation, syncytialization, and apoptosis of villous trophoblasts [1,2]. Thus, an increased RARRES1 expression in human PE might represent a state of reduced trophoblast proliferation and syncytialization with increased apoptosis [1,38,39].

In our current study, we determined that rodent Rarres1 was predominantly located in the placental junctional zone (giant cells and the spongiotrophoblast) with only minor expression in the labyrinth layer (resembling the human syncytiotrophoblast). Based on this observation, it could be assumed that Rarres1 plays a minor role in rodent placental syncytial physiology at the examined gestational stages. Other than a common hemochorial nature, murine placental anatomy shares limited features with the human placenta [40,41]. Nevertheless, the rodent placenta junctional zone (JZ) shares similarities with the human extravillous compartment [42,43,44,45], as it is positioned between the labyrinth and the maternal decidua [46]. From ~E12.5 onwards, trophoblast cells of the JZ invade into the decidua, where they become associated with maternal blood spaces. As a counterpart to the human placental syncytium, the JZ also constitutes the main placental endocrine compartment affecting both maternal and fetal physiology [46]. We have previously demonstrated that the placental distribution pattern of RARRES1 changes throughout human gestation [1]. Since we were able to detect temporal changes in the gestational expression of Rarres1 in mice, an involvement of Rarres1 in placental development and growth seems feasible, potentially regulating invasiveness of JZ trophoblast.

In contrast to our previous results from human PE placentas, *Rarres1* expression was not induced in placentas of eNOS^−/−^ mice. In fact, late gestational expression of placental *Rarres1* was rather reduced by dietary-induced IUGR in the rat. However, our *Rarres1* mRNA data were not supported by Western blot analysis, potentially owing to alterations in translation rate/protein degradation or transcription/mRNA stability (reviewed by [47]).

Our findings argue for a species-specific role of RARRES1 in the human syncytium and its involvement in PE [48,49]. While in the murine placenta temporal changes of placental *Rarres1* expression were noted, gestational changes in Rarres1 expression in the rat were not studied. No differences in Rarres1 expression were detected in placentas of male or female fetuses.

### 3.2. Expression of Rarres2 in Rodent Placenta

We found that our Wistar rats expressed Rarres2 in the same placental compartments as Rarres1 (see above). The pronounced localization of Rarres2 in rat trophospongium supports our previous findings in human placenta [1], where RARRES2 was specifically expressed in extravillous trophoblasts (see above).

The expression and regulation of Rarres2 during rat pregnancy has been previously studied by Garces et al. [6] in Sprague Dawley rats at multiple gestational timepoints. In contrast to our findings, Garces et al. [6] found relevant Rarres2 staining in the labyrinthine trophoblast, besides the trophospongium in rats, which might have been due to the difference in employed rat species (Wistar vs. Sprague Dawley). Similarly, they found syncytial RARRES2 expression besides its extravillous localization in the human placenta, which was not supported by our previous studies [1]. This difference requires further investigation. It might be due to divergent IHC techniques (fixation: methyl Carnoy’s solution (our study) versus paraformaldehyde [6], rabbit antibody vs. recombinant full length human RARRES2 (our study) versus goat antibody vs. N-terminal human RARRES2 [6]). The placental *Rarres2* expression increased until E16 in rats and then decreased until term, while rat maternal serum levels (ELISA) steadily decreased over the course of pregnancy in their animals [6]. This finding is in contrast to analyses of the same research group in humans, where RARRES2 levels were shown to rise significantly over the course of pregnancy [50]. This might argue for species-specific differences in the regulation of gestational Rarres2 expression and/or different functions of Rarres2 in murine and human placenta. The expression of *Rarres2* in rat mesenteric adipose tissue remained mainly constant, despite a singular increase at E19 [6]. IUGR (30% of total ad libitum maternal diet) resulted in a ~50% reduction of placental *Rarres2* expression in Sprague Dawley rats. The gestational expression pattern of *Rarres2* (i.e., maximum of placental expression around E16), however, remained unchanged [6]. Rarres2 expression in rodents was higher at the end of the second trimester, than at the end of the third trimester. This is in line with our findings of a more prominent Rarres2 expression in mouse placenta at gestational E15 compared to gestational day E18.5.

In contrast to Garces et al. [6], we did not observe a reduction of *Rarres2* in our isocaloric rat model of maternal protein restriction. This might be due to the divergent use of maternal diets (i.e., isocaloric protein restriction vs. total caloric reduction). We have just recently shown that our diet does not resemble a stress-model, unlike other models of total intake restriction [36]. Furthermore, models with total calorie restriction [51,52] seem more prone to develop insulin resistance after IUGR. Thus, the observed placental reduction of rat *Rarres2* expression under the condition of profound maternal food restriction [6] might underscore its proposed role as placental adipocytokine [6,53], with putative involvement in the development of maternal insulin resistance [54,55] and in feto-maternal metabolic homeostasis during pregnancy [56]. However, this hypothesis and the association of markers of insulin sensitivity with circulating RARRES2 is controversially discussed [50,57]. Unfortunately, there was no description of the influence of IUGR on *Rarres2* levels in rat maternal adipose tissue or serum by Garces et al. [6].

In vitro findings of Wang et al. [3] indicated that RARRES2 induces NO production in HUVECs. Our PE mouse model did not show local induction of *Rarres2* despite the knockout of eNOS, which suggests a lack of local negative feedback. At this point, systemic feedback-signaling cannot be ruled out, as circulating Rarres2 remained undetermined in our study.

### 3.3. Expression of CmklR1 in Rodent Placenta

Our finding of stable placental *CmklR1* expression levels in our Wistar rats resembled the gestational findings of Sanchez-Rebordelo et al. in Sprague Dawley rats [58]. Similarly, we could not detect significant differences or gestational changes of *CmklR1*-expression in our eNOS^−/−^ mice. This finding is of interest, as CMKLR1 activation has been shown to induce vasoconstriction of peripheral vessels [59] and a reduced uterine blood flow is characteristic in eNOS^−/−^ mice [60]. Thus, CmklR1 might play a minor role in the dysregulation of vascular tone in these mice. However, as knockout of CmklR1 seems to induce higher abortion rates in mice [61], different mechanisms of action need to be taken into consideration.

### 3.4. IL-11 as a Novel Regulatory Cytokine in eNOS^−/−^ Mice

The level of IUGR in our rats, as determined by fetal weight, was comparable to our previous experience with this model [36,62]. Additionally, the observed level of fetal and placental weight reduction in eNOS^−/−^ mice (18% and 22%, respectively; E18.5) was similar to findings from the literature (11% and 10%, respectively; E17 [28]), when compared to C57BL/6 control mice.

Interestingly, we found an induction of placental *IL-11* in our eNOS^−/−^ mice at midgestation, which has not been shown previously. As IL-11 has been demonstrated to contribute to the development of inflammation in PE and placental vascular changes in mice [24,25], our finding might indicate respective changes in our rodent placentas, which are found in a similar manner in human PE.

## 4. Materials and Methods

### 4.1. Animals and Diets

This study was carried out following the recommendations of the National Institute of Health (NIH) *Guide for the Care and Use of Laboratory Animals* and the Directive 2010/63/EU. All procedures and protocols were governmentally approved by the corresponding board (Regierung von Mittelfranken, AZ #54-2531.31-31/09 (10 November 2010) and AZ #55.2-2532-2-820 (17 January 2019)). Surgical procedures were performed under isoflurane anesthesia and all efforts were made to minimize suffering.

#### 4.1.1. Alimentary Rat Model with IUGR-Like Features

Animal procedures and the dietary regimen were carried out as previously described by us in detail [36]. Wistar rats were ordered from Charles River (Sulzfeld, Germany). Weighing 240–260 g, rats were mated, and the beginning of gestation was determined via assessment of vaginal plug expulsion. Subsequently, dams were randomly assigned into two groups consisting of six animals each and received semi-purified diets (Altromin Spezialfutter GmbH & Co. KG, Lage, Germany) of either low protein diet (LP group, 25 g/d of Altromin C1003, 8% protein) or an isocaloric diet of normal protein content (NP group, 25 g/d of Altromin C1000, 17% protein) from day 1 of gestation. This results in reduced birth weight and increased placental-to-fetal weight ratio, indicating preserved placental efficiency [63]. Rat placentas were obtained at E18.5. Animal characteristics are displayed in Table 1. Sex verification was carried out via sex-determining region Y (Sry) gene PCR, as previously described by us in detail [36].

#### 4.1.2. Mouse Model with PE/IUGR-Like Features

The eNOS-knockout (eNOS^−/−^) mice came from Jackson Laboratories (Bar Harbor, Maine, USA). The recommended wild-type (WT) C57BL/6 mice were ordered from Charles River (Sulzfeld, Germany). A homozygous breeding strategy was followed. Both strains were kept over ten generations in our animal facility before being utilized in experiments. Mice were housed at 22 ± 2 °C and a 12 h light/dark cycle in our animal facilities. Animals had unlimited access to standard chow (SSNIFF V1534, ssniff Spezialdiäten GmbH, Soest, Germany) and tap water. The animal model of eNOS^−/−^ mice was previously described in detail by others [28,64,65]. The placental dysfunction in eNOS^−/−^ mice [26,64,65] is caused with an impaired systemic vascularization of the dam [29]: eNOS deficiency significantly reduces the essential maternal cardiovascular adaptive capacity via reduction of circulating nitric oxide [28]. Thus, maintenance of constant uterine and feto-placental blood flow and of low feto-placental vascular resistance via modulation of smooth muscle myogenic tone is disabled [19,27,30]. Moreover, eNOS deficiency seems to be associated with an increased inflammatory state [31,32,33,34]. We chose eNOS^−/−^ mice over various other rodent models of PE (reviewed by [18]) as placentas of this model lack gross anatomic alterations [28] similar to our low protein rat model [66]. Each group of mice was mated, and the presence of a copulation plug was denoted as day 0.5 of pregnancy. Mouse placentas were obtained from these mice at day E15 and E18.5. Animal characteristics are displayed in Table 1. Based on our finding that sex seemed to have no influence on *Rarres1/2* expression in the rat placenta, we did not include it as a variable in the mouse model analysis. The placental *eNOS* mRNA expression was well detectable in control mice but was below the detection limit in eNOS^−/−^ mice (data not shown).

All rodent placentas were fixed in methyl Carnoy’s solution (Roth, Karlsruhe, Germany) for embedding in paraffin or were snap frozen and stored at −80 °C for messenger RNA (mRNA) preparation and protein extraction.

### 4.2. RNA Extraction, RT-PCR, and Real-Time Quantitative PCR

Gene expression analysis has previously been described by us in detail [36]. PCR was performed in *n* = 5 pups (mean) per litter from 4 NP/LP dams, respectively. In our mouse model, *n* = 2 pups per litter from *n* = 6 eNOS^−/−^ dams and *n* = 5 C57BL/6 wild type controls, respectively, were examined at two different time points (E15; E18.5). Snap-frozen placental tissues were minced using a Mikro-Dismembrator (Sartorius Stedim Biotech GmbH, Göttingen, Germany). RNA purification of our rat placentas was achieved with peqGold TriFast reagent (Peqlab, Erlangen, Germany), and RNA pretreatment with DNase I (Sigma-Aldrich, Darmstadt, Germany) was used. For mouse samples, the frozen tissue was homogenized by grinding with a T10 basic ULTRA TURRAX disperser (IKA, Staufen im Breisgau, Germany), and total RNA was extracted using the RNeasy Mini Kit with DNase treatment (Qiagen, Hilden, Germany) according to the manufacturer’s instructions. RNA concentration was determined by NanoDrop spectrophotometry (Peqlab, Erlangen, Germany) and adjusted to 100 ng/ mL for all rodent placenta samples. Complementary DNA (cDNA) synthesis was conducted using TaqMan Reverse Transcription (Applied Biosystems, Waltham, MA, USA) in a Biometra Trio thermal cycler (Analytik Jena, Jena, Germany). Quantification of *Rarres1*, *Rarres2*, *CmlkR1*, and *IL11* mRNA expression was achieved by qRT PCR analysis using the Fast SYBR Green Master Mix and Sequence Detector StepOnePlus (Applied Biosystems, Waltham, MA, USA) with *r18s* RNA as a reference gene. Measurements were performed in duplicate. Primers were designed using Primer Express software (version 3.0.1, Applied Biosystems, Waltham, MA, USA) or Primer-BLAST (NCBI, NIH). Primers were ordered from Eurofins (Eurofins Genomics Germany GmbH, Ebersberg, Germany) and sequences are listed in Table 4.

### 4.3. Western Blot Analysis

For protein expression analysis, placental tissue of rat NP/LP (4 dams with 2 pups/dam) and mouse E18.5 eNOS^−/−^ versus C57BL/6 control (8 dams with 1 pup each) was homogenized by mincing in 20 mL RIPA buffer, consisting of 50 mM Tris (pH 7.2), 10 mM EDTA, 150 mM NaCl, 0.1% SDS, 1.0% Triton X-100, 1.0% sodium deoxycholate, 20 μL/mL proteinase inhibitor (Complete proteinase inhibitor, Santa Cruz Biotechnology Inc., Dallas, TX, USA), and 2 mM Na3VO4. Buffer amount was adjusted to sample weight. The protein concentration was determined by the kit (Pierce, Rockford, IL, USA). Rat samples containing 30 µg/44 µl and mouse samples containing 30 µg/40 µL of protein were boiled at 95 °C for 8 min and separated on a 10% denaturing SDS-PAGE gel (for Rarres1 measurements of rat samples, 12% gel was used). Semi-dry electro-blotting was performed using Hartenstein GB33 PVDF membranes (Bio-Rad Laboratories, Hercules, USA), which were then blocked with Rotiblock (Roth, Karlsruhe, Germany) for 60 min. The membrane was incubated overnight at 4 °C with a polyclonal rabbit anti-rat antibody to Rarres1 (Biorbyt, Cambridge, UK) at a concentration of 1:250, or polyclonal rabbit anti-rat antibody to Rarres2 (Thermo Fisher, Waltham, MA, USA) at a concentration of 1:500 (rat)/1:1000 (mouse). Subsequently, the membrane was incubated for 60 min at room temperature with a secondary donkey anti-rabbit antibody (GE Healthcare, Amersham, UK) in the concentration 1:10,000 (for Rarres1 rat–blots 5% milk powder was added). As a reference, a monoclonal mouse anti-vinculin antibody at a concentration of 1:2000 and a monoclonal mouse anti-β-Tubulin antibody at a concentration of 1:10,000 (both from Sigma Aldrich, St. Louis, MO, USA) followed by a secondary sheep anti-mouse antibody (GE Healthcare, Amersham, UK) were used. As both reference genes resulted in similar results, only β-Tubulin blots were displayed. Immunoreactivity was visualized using the fluorescent ECL Plus Western Blotting Substrate according to the manufacturer’s instructions (Thermo Fisher Scientific, Waltham, MA, USA) and quantified with a luminescent imager (LAS-1000, Fujifilm, Berlin, Germany) and AIDA Image Analysis software (version 2.1, Elysia-raytest GmbH, Straubenhardt Germany).

Coomassie Brilliant Blue staining served as the loading control. 

### 4.4. Immunohistochemistry

For immunohistochemical (IHC) analysis, tissues were fixed in methyl Carnoy’s solution and embedded in paraffin, as previously described [67]. Each group consisted of 6 placentas (3 sections of the central region, each) from 2 dams. Two-micrometer paraffin sections were prepared with a HM340E microtome (Thermo Fisher Scientific, Waltham, MA, USA). After de-paraffinization and rehydration with intermittent Tris-buffered saline (TBS) washing, tissue sections were unmasked by cooking in target retrieval solution (TRS, Dako Agilent, Santa Clara, CA, USA) for 10 min. Endogenous peroxidase activity was blocked with 3% H2O2 for 20 min at room temperature. Sections were then incubated in fetal calf serum (FCS) at 37 °C for 30 min and coated with the primary antibody (Rarres1: MyBioSource, San Diego, CA, USA, 1:50; Rarres2: Thermo Fisher, Waltham, MA, USA 1:100). After incubation at 4 °C overnight, sections were washed in TBS and layered with the secondary antibody (dilution 1:500; biotin-conjugated, goat anti-rabbit immunoglobulin G; Vector Laboratories, Burlingame, CA, USA) at room temperature for 30 min. Subsequently, sections were incubated with avidin-biotinylated horseradish peroxidase complex (Vectastain PK-6100; Vector Laboratories) at RT for 30 min and with a DAB (diaminobenzidine tetrahydrochloride) kit (SK-4100; Vector Laboratories, both supplied by Linaris, Dossenheim, Germany) for 15 min and counterstained with hematoxylin (Merck, Darmstadt, Germany). After embedding in Entellan (Merck, Darmstadt, Germany), imaging was performed with a DMC 6200 camera mounted on a Leica DMR microscope (Type 020-525.731) using LASX 3.4.2.18368 image software (all from Leica Microsystems, Wetzlar, Germany). Representative photomicrographs for antibody specificity testing are shown in supplementary Figure S1.

### 4.5. Statistical Analysis

Results were expressed as mean ± standard deviation (SD) unless stated otherwise. Statistical analyses were performed using GraphPad Prism software (version 7.0, GraphPad Software, San Diego, CA, USA). We checked for outliers by using the PRISM “robust regression and outlier removal” (ROUT) method (Q = 1%, equivalent to a false discovery rate of 1%), as described by Motulsky and Brown [68] and Hughes and Hekimi [69]. Excluded data points (*n* = 0 in rats; *n* = 1 for mouse PCR of *Rarres1*, *CmklR1*, and *IL-11*, respectively) were not included in the calculation of the mean per litter. Subsequently, the means per litter were subjected to further statistical analysis. Before performing groupwise comparisons, outliers were removed [36] and a non-parametric Mann–Whitney U-test was executed. A *p* value <0.05 was considered statistically significant. Data processing and imaging was performed with Microsoft Office 2016 (Microsoft, Redmond, WA, USA) and Adobe Photoshop CS6 (Adobe Systems, San José, CA, USA).

## 5. Limitations

In our study, rodent placental tissue was analyzed in toto. Thus, compartment specific changes might have been masked. We did not analyze circulating Rarres2 levels in maternal or fetal serum. Thus, at this point, our conclusions regarding Rarres1/2 are limited to the placental level only. In line with this limitation, no other local sources of Rarres1/2 (e.g., adipose tissue) were evaluated in our study and only certain gestational time-points were examined. Thus, temporal changes in placental expression profiles remain elusive. The choice to analyze mid-/late-gestational placental tissue was based on our previous findings in human third trimester placentas and trophoblasts [1,2]. Consequently, a potential involvement of Rarres1/2 in placentation and early gestation of our animal models remains to be determined. Furthermore, the use of eNOS^−/−^ as a model for IUGR or preeclampsia has been controversially discussed [70,71]. This model is characterized by impaired endothelial function with uterine artery dysfunction and a lack of blood vessel expansion, as well as a placental transport phenotype [26]. Therefore, eNOS^−/−^ might only represent certain early subtypes of human PE and/or IUGR, which on the other hand may not be relevant to rodents themselves.

## 6. Conclusions

To our knowledge, we were the first to examine Rarres1 localization in the rodent placenta. Also, Rarres1 and 2 expressions have not been studied in the above rodent models.

Rarres1/2 findings in both animal models did not resemble placental alterations of RARRES1/2 observed by us in human PE or PE/IUGR. These results might indicate species-specific differences in placental regulation and compartmentation. The fact that others observed reduced placental Rarres2 expression following more profound maternal food restriction suggests metabolic functions of the peptide beyond its potential tumor-suppressor role that need further investigation. Furthermore, the clarification of a potential feto-maternal crosstalk via adipocytokine Rarres2 and its possible role in the regulation of immunologic and inflammatory processes at the placental interface requires further functional studies. Moreover, the role of IL-11 in the placental pathophysiology of eNOS^−/−^ mice remains to be determined.

## Figures and Tables

**Table 1:** Animal auxology. NP: normal protein diet; LP: low protein diet.

**Rat ^†^**	**E18.5 NP**	**E18.5 LP**	***p* Value**			
fetal weight (fw)	1.38 ± 0.09	0.86 ± 0.05	0.03 *			
placental weight (pw)	0.34 ± 0.04	0.30 ± 0.01	0.11 *			
pw/fw ratio	0.25 ± 0.02	0.35 ± 0.02	0.03 *			
**Mouse ^‡^**	**E15 C57BL/6**	**E15 eNOS^−/−^**	***p* Value**	**E18.5 C57BL/6**	**E18.5 eNOS^−/−^**	***p* Value**
fetal weight (fw)	0.34 ± 0.07	0.28 ± 0.03	0.08 *	1.19 ± 0.15	0.97 ± 0.08	0.006 *
placental weight (pw)	0.10 ± 0.01	0.08 ± 0.01	<0.001 *	0.09 ± 0.01	0.07 ± 0.01	<0.001 *
pw/fw ratio	0.31 ± 0.06	0.29 ± 0.06	0.58 *	0.08 ± 0.02	0.07 ± 0.01	0.63 *

* Mann-Whitney U-Test. ^†^ For rats, each group consisted of n = 4 dams each with n = 6 NP/LP pups/damn, respectively. ^‡^ For mice, groups consisted of n = 6 eNOS^−/−^ vs. n = 5 C57BL/6 dams at both time points with n = 2 pups/dam. Legend: bold values denote statistical significance.

**Table 2:** Sex differences in mRNA expression (fold-change).

Rat	NP m	NP f	*p* Value	LP m	LP f	*p* Value
*Rarres1*	1.00 ± 0.36	0.97 ± 0.17	0.89 *	0.48 ± 0.26	0.58 ± 0.19	0.69 *
*Rarres2*	1.00 ± 0.30	1.30 ± 0.41	0.49 *	0.90 ± 0.75	1.52 ± 0.42	0.23 *
*CmklR1*	1.00 ± 0.44	0.89 ± 0.60	0.99 *	1.10 ± 0.51	0.95 ± 0.29	0.99 *

* Mann-Whitney U-Test; For Rarres1 and 2, groups consisted of n = 4 NP/LP dams with n = 3 female/male pups, respectively. For CmklR1, groups consisted n = 3 NP/LP dams with n = 2 female/male pups, respectively. Abbreviations: LP = low protein diet, NP = normal protein diet in the rat IUGR model; m = male fetus, f = female fetus.

**Table 3:** Placental *CmklR1* mRNA expression (fold-change).

**Rat ^†^**	**E18.5 NP**	**E18.5 LP**	***p* Value**			
	1.00 ± 0.53	1.08 ± 0.32	0.99 *			
**Mouse ^‡^**	**E15 C57BL/6**	**E15 eNOS^−/−^**	***p* Value**	**E18.5 C57BL/6**	**E18.5 eNOS^−/−^**	***p* Value**
	1.00 ± 0.43	0.9 ± 0.42	0.93 *	0.51 ± 0.1	0.41 ± 0.18	0.18 *

* Mann-Whitney U-Test. ^†^ For rats, groups consisted of n = 3 dams per group (NP/LP) with n = 5 pups each; ^‡^ For mice, groups consisted of n = 6 eNOS^−/−^ vs. n = 5 C57BL/6 dams at both time-points with n = 2 pups/dam. Abbreviations: LP = low protein diet, NP = normal protein diet in the rat IUGR model; C57BL/6 = Wild type (WT) control, eNOS^−/−^ = PE/IUGR model knockout mouse.

**Table 4:** Primer sequences.

**Rat**	**Forward**	**Reverse**
*Rarres1*	5′-AGGTGGACCTGGTGTTTAGCA-3′	5′-AACACCCTCGCAGAACATTTG-3’
*Rarres2*	5′-AAATGGGAGGAAGCGGAAAT-3′	5′-CCATCCGGCCTAGAACTTTACC-3′
*CmlkR1*	5′-AAGAGATGGAGTACGAGGGTTACAA-3′	5′-GATGTAGTCCGAGCCGTCAGA-3′
*r18s*	5′-TTGATTAAGTCCCTGCCCTTTGT-3′	5′-CGATCCGAGGGCCTCACTA-3′
**Mouse**		
*Rarres1*	5′-AGCGGCTGAAAACGGATGA-3′	5′-CCAAGTGAATACGGCAGGGA-3′
*Rarres2*	5′-CACTGCCCAATTCTGAAGCAA-3′	5′-CGCCAGCCTGTGCTATCTTAA-3′
*Cmlkr1*	5′- CAACGGTGAACAGTGAAAGGTC-3‘	5′- TTGTAAGCGTCGTACTCCATCTCT-3‘
*eNos*	5′-CACCAGGAAGAAGACCTTTAAGGA-3′	5′-CACCGTGCCCATGAGTGA-3′
*IL-11*	5′-GCTCCCCTCGAGTCTCTTCA-3′	5′-TGTCTCTCATCTGTGCAGCTAGTTG-3′
*r18s*	5′-TTGATTAAGTCCCTGCCCTTTGT-3′	5′-CGATCCGAGGGCCTCACTA-3′

## Supplementary Materials

The following are available online at https://www.mdpi.com/1422-0067/21/1/242/s1, Figure S1: Specificity testing of the antibody to RARRES1, Figure S2: Specificity testing of the antibody to RARRES2.
