# Supplementary material for: Expression of Retinoid Acid Receptor-Responsive Genes in Rodent Models of Placental Pathology
Source: Int J Mol Sci. 2019 Dec 29;21(1):242. doi: 10.3390/ijms21010242 (PMC6981780; doi:10.3390/ijms21010242)
Supplement: Supplementary file 1 [file ijms-21-00242-s001.pdf]

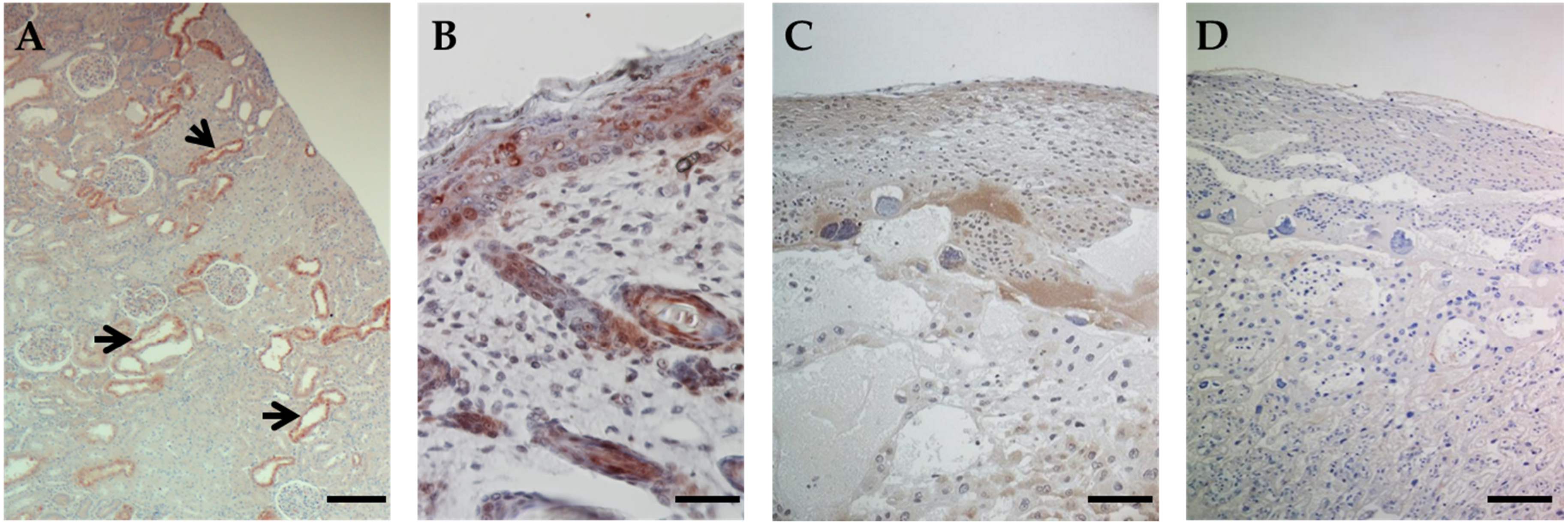

Figure S1. Specificity testing of the antibody to RARRES1. (A) Positive control staining: RARRES1 in rat kidney tubuli (black arrows), as also described in: https://www.proteinatlas.org/ENSG00000118849-RARRES1/tissue, scale bar = 100 μm. (B) RARRES1 staining of rat skin, scale bar = 50 μm. (C) RARRES1 staining of rat placenta, scale bar = 100 μm. (D) Negative control staining of rat placenta using diluted pre-immune serum, scale bar = 100 μm.

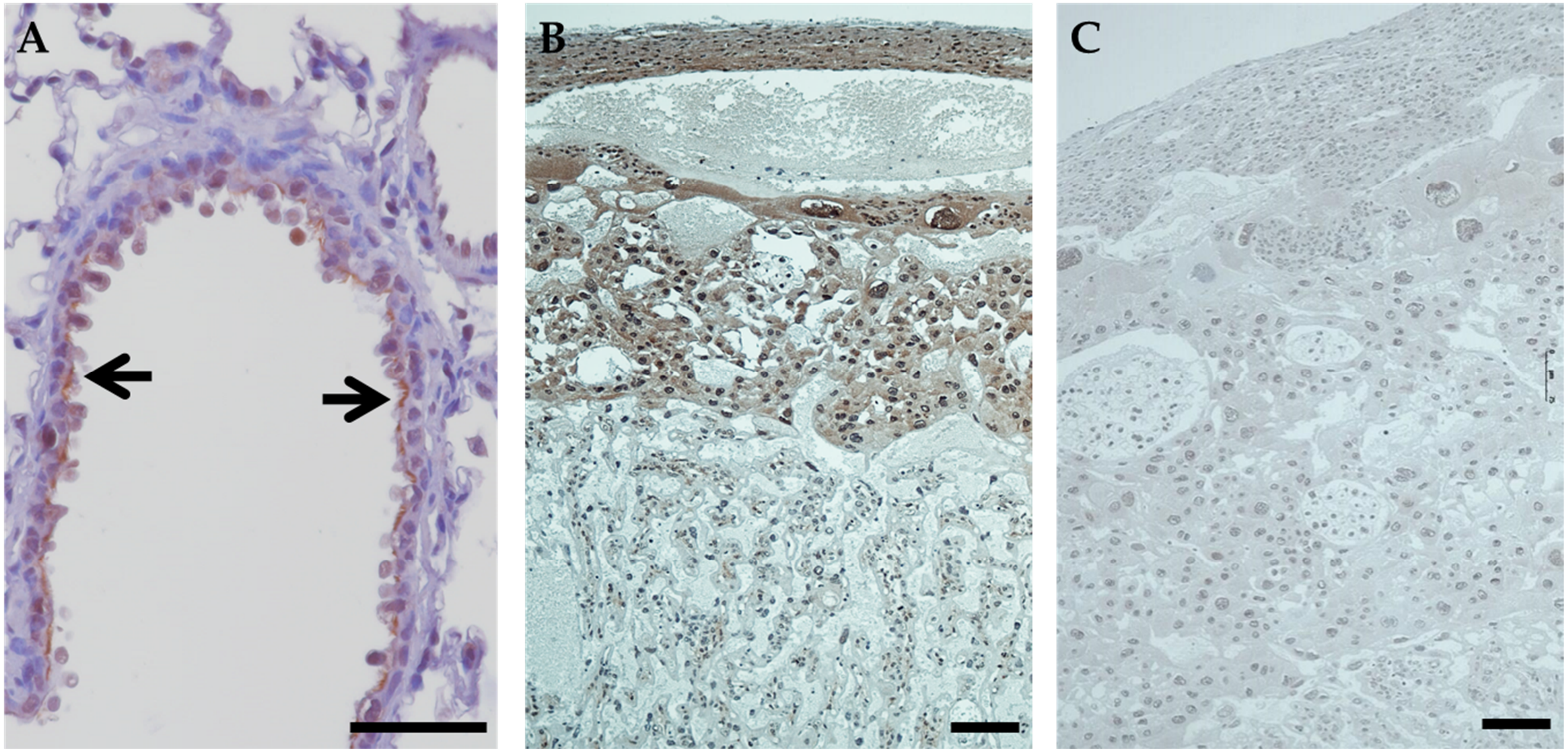

Figure Figure 2. Specificity testing of the antibody to RARRES2. (A) Positive control staining: RARRES2 in rat lung, as also described by [1], scale bar = 50 μm. (B) RARRES2 staining of rat placenta, scale bar = 100 μm. (C) Negative control staining of rat placenta using diluted pre-immune serum, scale bar = 100 μm.

References

1. Demoor, T.; Bracke, K.R.; Dupont, L.L.; Plantinga, M.; Bondue, B.; Roy, M.O.; Lannoy, V.; Lambrecht, B.N.; Brusselle, G.G.; Joos, G.F. The role of ChemR23 in the induction and resolution of cigarette smoke-induced inflammation. *J Immunol*. **2011** *186*, 5457–5467.
